# Insights into the Function of Long Noncoding RNAs in Sepsis Revealed by Gene Co-Expression Network Analysis

**DOI:** 10.3390/ncrna3010005

**Published:** 2017-01-26

**Authors:** Diogo Vieira da Silva Pellegrina, Patricia Severino, Hermes Vieira Barbeiro, Heraldo Possolo de Souza, Marcel Cerqueira César Machado, Fabiano Pinheiro-da-Silva, Eduardo Moraes Reis

**Affiliations:** 1 Programa Interunidades de Pós-Graduação em Bioinformática, Universidade de São Paulo, 05508-090 São Paulo, Brazil; diogo@iq.usp.br; 2 Instituto Israelita de Ensino e Pesquisa, Hospital Israelita Albert Einstein, 05652-900 São Paulo, Brazil; patricia.severino@einstein.br; 3 Departamento de Emergências Clínicas, Faculdade de Medicina da Universidade de São Paulo, 05403-000 São Paulo, Brazil; hermes.barbeiro@fm.usp.br (H.V.B.); heraldo.possolo@fm.usp.br (H.P.d.S.); mccm37@uol.com.br (M.C.C.M.); pinheirofabiano@hotmail.com (F.P.-d.-S.); 4 Departamento de Bioquímica, Instituto de Química, Universidade de São Paulo, 05508-000 São Paulo, Brazil

**Keywords:** sepsis, aging, inflammation, transcriptome, long noncoding RNAs, co-expression networks

## Abstract

Sepsis is a major cause of death and its incidence and mortality increase exponentially with age. Most gene expression studies in sepsis have focused in protein-coding genes and the expression patterns, and potential roles of long noncoding RNAs (lncRNAs) have not been investigated yet. In this study, we performed co-expression network analysis of protein-coding and lncRNAs measured in neutrophil granulocytes from adult and elderly septic patients, along with age-matched healthy controls. We found that the genes displaying highest network similarity are predominantly differently expressed in sepsis and are enriched in loci encoding proteins with structural or regulatory functions related to protein translation and mitochondrial energetic metabolism. A number of lncRNAs are strongly connected to genes from these pathways and may take part in regulatory loops that are perturbed in sepsis. Among those, the ribosomal pseudogenes *RP11-302F12.1* and *RPL13AP7* are differentially expressed and appear to have a regulatory role on protein translation in both the elderly and adults, and lncRNAs MALAT1, LINC00355, MYCNOS, and AC010970.2 display variable connection strength and inverted expression patterns between adult and elderly networks, suggesting that they are the best candidates to be further studied to understand the mechanisms by which the immune response is impaired by age. In summary, we report the expression of lncRNAs that are deregulated in patients with sepsis, including subsets that display hub properties in molecular pathways relevant to the disease pathogenesis and that may participate in gene expression regulatory circuits related to the poorer disease outcome observed in elderly subjects.

## 1. Introduction

Sepsis is a major cause of death in the world and novel mechanisms of bacterial resistance and virulence are further increasing its incidence in intensive care units. Despite the efforts of the scientific community, the molecular mechanisms associated to its pathogenesis remain poorly understood. Evidences obtained through high-throughput gene expression analysis have revealed sustained upregulation of genes related to innate immunity and the concomitant downregulation of adaptive immunity genes in the blood of septic patients [1]. These results suggest that the classic biphasic model of overall proinflammatory signaling, named SIRS (systemic inflammatory response syndrome), followed by overt immunodepression, named CARS (compensatory antagonistic response syndrome) is controversial, indicating that the molecular basis of sepsis is more complex than anticipated [2].

The incidence of sepsis increases exponentially with age, and older age is an independent risk factor for mortality among adults hospitalized with sepsis [3]. Global gene expression studies of innate immunity cells have shown that impairment of mitochondrial function significantly contributed to organ failure in septic patients [4] and that a marked decrease in the expression of genes encoding components of the mitochondrial respiratory chain occurs in the septic elderly [5].

Most gene expression studies in sepsis have focused on protein-coding genes and generally overlooked the expression patterns of noncoding RNAs, which comprise different classes of molecules that are not translated into proteins. Operationally, noncoding RNAs can be broadly divided in two major classes based on their length: small RNAs (<50 nt) and long (>200 nt) noncoding RNAs (lncRNAs) [6]. MicroRNAs comprise a class of well-known small (21–23 nt) ncRNAs that act through the post-transcriptional regulation of their mRNA targets, either by mRNA destabilization of translational repression [7]. Several microRNA signatures have already been reported in septic patients [8,9,10]. Ma et al. described that miR-150 and miR-4772-Sp-iso are able to discriminate septic patients from those affected by other causes of systemic inflammation [8], and Vasilescu et al. found miR-150 as a prognostic marker in patients with sepsis [10]. Tacke et al., moreover, identified elevated levels of miR-133a in serum from septic patients [9] and Wang et al. demonstrated that miR-27a is upregulated in lungs of septic mice and regulates the inflammatory response [11].

Conversely, the role of lncRNAs in sepsis has not been investigated in detail. Thousands of lncRNAs have been identified in multiple species [12]. It is an ongoing debate whether all of the transcriptional activity that produces long noncoding RNAs serves important biological functions, but it has become evident that changes in the expression levels of many lncRNAs are correlated with several developmental and disease states, including cancer [13,14]. Detailed biochemical and functional studies have determined a variety of novel mechanisms of gene expression regulation mediated by lncRNAs [15,16]. As an example, lncRNAs may regulate gene expression by recruiting chromatin and histone modifiers to specific genomic sites, thus causing transcriptional gene repression or activation [17]. In addition to regulating DNA transcription, lncRNAs have been shown to modulate post-transcriptional processes such as alternative splicing, nuclear trafficking, mRNA stability, and translation [6,18,19].

Gene co-expression networks are useful to represent functional associations amongst components of the cellular transcriptome in different experimental conditions [20]. It is expected in biological systems that some genes are to be more connected than others, acquiring a hub behavior (“hubbyness”), and when this gene is an lncRNA, it can be hypothesized that it acts as a regulator of other genes to which it is significantly correlated [21].

In this study, we performed a global analysis of lncRNA expression in neutrophil granulocytes from septic patients, both adults and elderly, compared to healthy controls. We observed hundreds of lncRNAs from different classes (intergenic, antisense, and intronic lncRNAs) that are deregulated in patients with sepsis. Among these, we found subsets of lncRNAs that display hub properties in molecular pathways previously shown to be preferentially perturbed in elderly individuals and, therefore, may have regulatory roles that contribute to the worse disease outcome in this group of patients.

## 2. Results

### 2.1. Identification of RNA Expression in the Innate Immune System in Sepsis

To take advantage of the most recent lncRNA compendia, the probes from the commercial Agilent oligoarray were filtered and reannotated according to updated gene annotation databases (GENCODE, Broad Institute Human lincRNA, LNCipedia, NONCODE). Following genome mapping, filtering of multi-aligning probes, and cross-reference with catalogs of protein-coding and noncoding RNAs (see Materials and Methods for details) a total of 47,012 array probes were approved and assigned to protein-coding mRNAs (26,542), lncRNAs (14,832), and pseudogenes (2869). Probes that could not be unambiguously associated to a known RNA annotation (2781) were labeled as “poorly annotated RNAs” (Table 1, Figure S1).

The reannotated probes assessed the expression of neutrophil-enriched granulocyte fractions from 24 subjects, including young adults (*n* = 12) and elderly (*n* = 12) subjects. Each age group comprised an equal number of healthy controls and sepsis patients. An additional quality filtering excluded those with intensity signals near the background values (see Materials and Methods for details). A total of 11,895 protein-coding mRNAs, 834 pseudogenes and 1185 lncRNAs were detected in at least one sample group and were further analyzed (Table 1).

**Figure 1:**
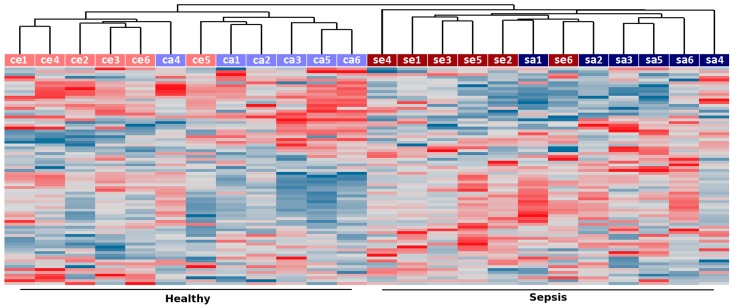
Unsupervised hierarchical clustering of samples from sepsis (“se” for elderly and “sa” for adults) and healthy controls (“ce” for elderly and “ca” for adults) based on long noncoding RNA (lncRNA) expression. Expression measurements from the top 5% more variable lncRNAs (based on their coefficient of variation across all samples) were used to group samples using UPGMA clustering and Pearson correlation as a distance measurement. Samples are labeled according to disease status (sepsis in red, healthy controls in blue) and age (elderly subjects are shown in darker colors whereas those from young adults are shown in lighter colors).

As shown in Figure 1, unsupervised clustering of the 5% most variable lncRNAs (77 transcripts) correctly grouped the samples according to disease status, indicating the existence of coordinated changes in the expression of lncRNAs in neutrophil granulocytes from septic patients. Noteworthy, samples from septic and control subjects showed a trend to cluster adult and elderly samples, suggesting that the expression of noncoding RNAs is also affected by age (Figure 1). As previously reported [5], over a thousand protein-coding genes (≈10% of detected) displayed changes in transcript abundance in septic patients compared to age-matched controls (Table 1). Here, we found that a comparable fraction of lncRNAs (≈9% of detected) are significantly changed in sepsis (*p* value ≤ 0.01) both in the adults and elderly patients (Table 1). A list with all detected probes along with their gene type (protein-coding, lncRNA, pseudogene, poorly annotated), gene ID, chromosomal location, relative expression between septic and control samples and associated *p* values is provided as Supplementary Material (Table S1).

Two hundred and ten lncRNAs were differentially expressed in sepsis (*p* ≤ 0.01; Table 1, Table S1). We asked whether these lncRNAs could be affecting the expression of neighboring protein-coding genes. We searched for the nearest protein-coding gene in either direction, and for 170 lncRNAs, the neighboring protein-coding gene was also interrogated in the array. From these, only 88 were measured above the detection threshold and 55 where differently expressed in sepsis. For each lncRNA–protein coding pair, we calculated the expression correlation among all samples. We found that the measured correlations were not significant (Bonferroni-corrected *p* value > 0.01) compared to the distribution of correlations measured for all measured gene pairs. A similar result was obtained using only 39 lncRNA–protein coding pairs for which the neighboring genes was at a distance shorter than 4000 bp. We did not find any functional gene category or molecular pathway enriched among the lists of neighboring protein coding gene sets described above. Altogether, these results do not favor the notion that the lncRNAs deregulated in sepsis act in *cis* to affect the expression of neighboring genes.

Next, to gain insights on the possible functions of lncRNAs in sepsis, we investigated their pattern of co-expression with protein-coding genes in samples from adult and elderly patients.

### 2.2. Co-Expression Networks of mRNAs and lncRNAs Are Perturbed in Sepsis

**Figure 2:**
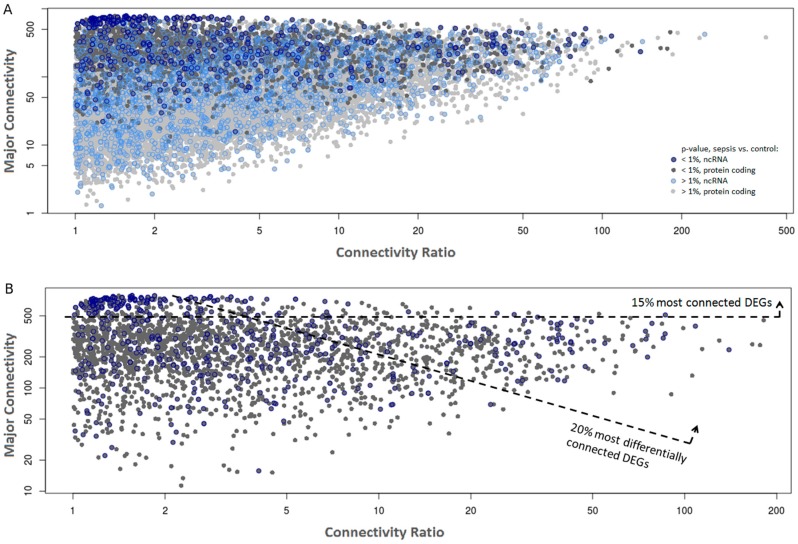
Gene co-expression networks from sepsis and control samples. Each dot represents a gene plotted as a function of its major network connectivity (*y*-axis) and the connectivity ratio, which is the ratio between the major connectivity/minor connectivity retrieved from the adult and elderly networks (*x*-axis). ncRNAs are marked with a blue circle. (**A**) All detected genes colored by differential expression significance between sepsis and control samples, with differentially-expressed genes (DEGs) indicated by a darker shade. (**B**) Only DEGs (*p* ≤ 0.01) are shown. The most connected (top 15%) and most differentially connected (top 20%) genes are highlighted. The top 20% most differentially connected DEGs were selected using the product of the average connectivity in both networks and the connectivity ratio across networks as a cutoff.

Two separate gene co-expression networks (adult and elderly networks) were constructed using the WGCNA package [22] using all genes that passed the filtering step (Figure S2). Next, the connectivity value measured for each gene in each network was retrieved and the highest value (“major connectivity”, Figure 2, *y* axis) was plotted as a function of the connectivity ratio, which is the ratio between the major connectivity/minor connectivity retrieved from the adult and elderly networks (“connectivity ratio”, Figure 2, *x* axis). Data from all transcripts that passed the filters (Figure 2A) or only those that were differentially expressed between septic and control samples (Figure 2B) are shown. We observed that genes differentially expressed in sepsis are significantly more connected than stably expressed genes (*p* < 10^−10^), with differentially expressed genes (DEGs) showing average connectivity of 300 (SD = 188) and non-DEGs 112 (SD = 118), suggesting that these may have greater influence in the establishment of the co-expression networks in the innate immune system both in adults and in the elderly.

To gain insight into the most functionally relevant components of the networks, we focused our analysis on the subset of DEGs in sepsis (sepsis vs controls, *p* ≤ 0.01; Figure 2B). Among these, we arbitrarily sub-selected the most connected (top 15%, *n* = 385) and the most differentially connected genes (top 20%, *n* = 514) across the adult/elderly networks. With these criteria, we aimed to favor the selection of genes that are central to sepsis regulation (top 15% most connected) or genes that are differentially perturbed in sepsis according to the age of the subject (top 20% most differentially connected), respectively.

**Figure 3:**
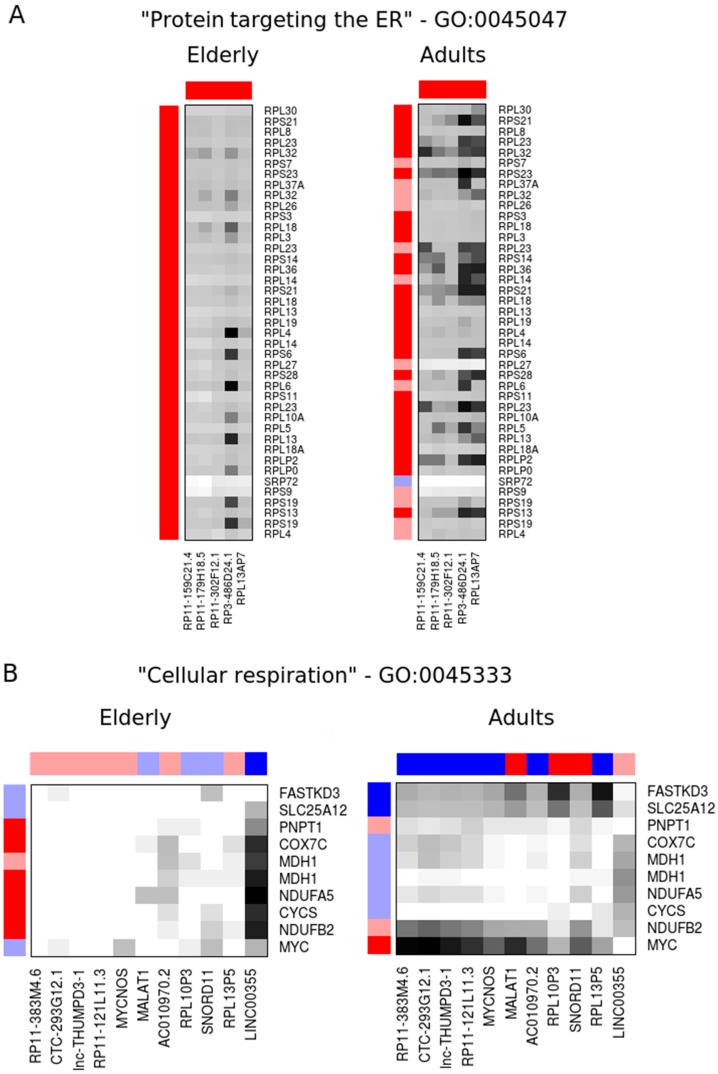
Elderly and adult co-expression networks of selected lncRNAs (columns) and protein-coding mRNAs (rows) in molecular pathways enriched among the most connected (**A**) or the most differentially connected (**B**) genes. Heatmap colors show the network similarity between each gene pair, with black being the most similar and white the least similar. The gene expression ratios are shown as external bars, where dark blue and red indicate transcripts significantly upregulated or downregulated in sepsis relative to controls (*p* ≤ 0.01), respectively.

We postulate that the lncRNAs ranked among the top most connected/top most differentially connected transcripts might participate in the same pathways or share regulatory mechanisms with the protein-coding gene to which they are connected. Thus, we used the gProfiler tool to evaluate the enrichment of specific terms among the most connected genes. We found that the top 15% most connected genes are over-represented, with terms related to protein translation such as “peptide chain elongation” (REAC:156902, *p* = 2 × 10^−16^), “cytosolic ribosome” (GO:0022626, *p* = 4 × 10^−22^), and “protein targeting to the endoplasmic reticulum” (GO:0045047, *p* = 7 × 10^−22^). These enriched sets have a significant overlap (>90%) and predominantly encode ribosomal proteins (Figure S3A). From the 112 lncRNAs present in the top 15% most connected genes, there are five lncRNAs (RP11-159C21.4, RP11-179H18.5, RP11-302F12.1, RP3-486D24.1 and RPL13AP7) that showed an average local similarity above the median similarity of the enriched genes (as described in Materials and Methods) and are also deregulated in sepsis in the same direction (i.e., up- or down-regulated) both in elderly and adult co-expression networks (Figure 3A and Table 2).

Likewise, the biological process term “cellular respiration” (GO:0045333, *p* = 0.049) was over-represented among the top 20% most differentially connected genes (Figure S3B). Within the 89 lncRNAs present in this set, we note that 11 (RP11-383M4.6, CTC-293G12.1, lnc-THUMPD3-1, RP11-121L11.3, MYCNOS, MALAT1, AC010970.2, RPL10P3, SNORD11, RPL13P5 and LINC00355) displayed local network similarity above the median in at least one network while showing an inverted expression pattern in sepsis between the adult and elderly networks (i.e., upregulated in one network while downregulated in the other) (Figure 3B and Table 3).

The genomic loci encoding lncRNAs differentially expressed in sepsis and displaying high connectivity in gene co-expression networks display evidence of evolutionary sequence conservation and contain putative regulatory DNA elements (promoter-associated H3K4me3, enhancer-associated H3k27ac, transcription factor binding sites) (Figure S4A–H).

## 3. Discussion

Despite numerous studies investigating the role of lncRNAs in various diseases, their roles in the innate immune system during infection are only now emerging [23,24]. The lncRNAs are widely expressed in immune cells during their development, differentiation, and activation, and they can also control important aspects of immunity [25]. The lincRNA-Cox2, for example, is highly induced by numerous inflammatory triggers and interferes with NF-κB signaling [26], while the lncRNA Lethe, a functional pseudogene, physically binds to p65 in mouse embryonic fibroblasts (MEFs), inhibiting its occupancy at the promoter of target genes, such as interleukins 6 and 8 (*IL6* and *IL8*) [27], and THRIL controls the expression of tumor necrosis factor α (TNFα) in the human monocyte-like THP-1 cell line [28].

In this work, we report the global gene expression analysis of neutrophil-enriched cell fractions from patients with sepsis and age-matched controls, focusing on the noncoding component of the transcriptome. The expression data was previously generated using a commercially available Agilent oligoarray platform [5], and we initially performed a probe reannotation procedure to take advantage of the most updated lncRNA information available in public domain. This updated annotation (GEO entry GPL22628) will allow researchers to revisit publicly available expression data sets and perform original analyses focused on lncRNAs. Following this procedure, we identified over 1000 lncRNAs, including pseudogenes, that are detected in neutrophil-enriched samples, a fraction of which display differential abundance in septic patients compared to control subjects. For the most part, the biological processes in which these lncRNAs participate are unknown. We did not observe any significant association between the expression of lncRNAs and neighboring protein-coding genes differentially expressed in sepsis. To highlight lncRNAs presumably relevant in the context of sepsis, we incorporated information from co-expressed protein-coding genes. This approach can indicate *trans*-acting regulatory lncRNAs. We found that the most differentially expressed transcripts are also among the most connected in the sepsis gene expression networks. Furthermore, we observed that the most connected DEGs are enriched in gene categories encoding protein components of ribosomes, protein synthesis, and localization. We raise the possibility that the lncRNAs with most network similarity to these genes are potentially involved in regulatory circuits associated to ribosomal components that are deregulated in sepsis. Thus, we identified the lncRNAs RP11-159C21.4, RP11-179H18.5, RP11-302F12.1, RP3-486D24.1, and RPL13AP7 as candidates to be further investigated as biomarkers for sepsis (Figure S4A–E). Interestingly, all of those are transcribed from pseudogenes related to the ribosomal proteins RPS13, RPS8, RPS29, RPL7A, and RPL13A, respectively. We think this common ancestry further supports the idea that these pseudogene-associated lncRNAs have a functional or regulatory role in protein translation.

Immunosenescence affects many components of the immune system, and sepsis is a disease of older people [29]. Indeed, 60% of all sepsis events and 80% of septic deaths occur in individuals over 65 years old [30]. Most studies comparing changes in the immune system from septic patients of advanced age with young adults have evaluated changes in cellular and humoral components of the immune response [31]. Few studies have investigated changes in elderly septic patients using global gene expression profiling [5]. To investigate how aging affects the immune response of the elderly, we selected genes with high connectivity in either the adult or elderly network. The protein-coding genes in this set were enriched for terms associated to “cellular respiration”. Among the most connected genes in this set, we found *MYC*, which is a positive regulator of mitochondrial biogenesis and metabolism [32], and *FASTKD3*, which modulates energy balance in stress conditions by functionally coupling mitochondrial protein synthesis to respiration [33]. It also includes genes encoding components of the mitochondrial electron transport chain (*CYCS, NDUFB2, NDUFA5, COX7C*) or involved in transport across the mitochondrial membrane (*MDH1, SLC25A12, PNPT1*). All these genes (exception of *COX7C*) are downregulated in adult and elderly septic patients. This observation is consistent with a previous study, which found that genes related to oxidative phosphorylation and mitochondrial dysfunction are preferentially deregulated in the elderly with sepsis [5]. Mitochondria are the respiratory and energetic centers of cells. However, mitochondrial dysfunction enhances reactive oxygen species (ROS) production [34]. ROS are highly unstable structures that cause cell damage. Oxidative stress results when ROS production and the antioxidant protection mechanism are imbalanced [35]. Mitochondrial function in sepsis is highly variable, organ-specific, and predicts a worse outcome [36]. Inhibition of oxidative phosphorylation results in a reduction of the mitochondrial membrane potential, and consequently a lack of energy, which can cause organ failure and death [37,38].

Here, we raise the hypothesis that lncRNAs that show an inverted expression pattern in sepsis and are also differentially connected across elderly and adult networks could participate in gene expression regulatory loops that potentiate the loss of mitochondrial function in the elderly with sepsis. These include AC010970.2, MYCNOS, LINC00355, and MALAT1 (Figure S4F–H) that will be mentioned further. MALAT1 is upregulated in various tumors and has oncogenic roles [16]. MALAT1 has been implicated in the positive regulation of inflammatory processes induced by hyperglycemia [39], but its participation in sepsis has not been documented before. Our data suggest that reduced levels of MALAT1 may contribute to gene expression changes associated to the poorer outcome of elderly patients. MYCNOS is an lncRNA known to function as an antisense RNA that regulates MYCN, a member of the MYC family of transcription factors [40]. There is little information available regarding the two other lncRNAs; LINC00355, the only one of the selected genes from Table 3 to be highly connected and differentially expressed in the elderly, is a lincRNA that was not previously studied in the literature, and AC010970.2 is an 18S ribosomal pseudogene.

We note that our study is exploratory and employed a limited sample size, thus future functional studies will be essential to determine the biological significance of lncRNAs in sepsis and to dissect their mechanisms of action. Our future plans include the investigation of lncRNAs in other cell types and tissues during sepsis, such as in the central nervous system. The treatment of sepsis lacks effective specific drugs. A recent review of the current experimental treatments of mitochondrial dysfunction in sepsis has been published, and in animal experiments many drugs show good results [41], but clinical trials still wait to be done, especially in older patients.

In summary, here we report lncRNAs with aberrant expression in sepsis, including subsets that are significantly co-expressed with protein-coding genes from molecular pathways relevant to the disease, and that are potentially associated to the worse outcome observed in aged subjects. Further experimental studies are warranted to investigate the clinical relevance of these lncRNAs for the development of novel biomarkers or new therapeutic strategies for the disease.

## 4. Materials and Methods

### 4.1. Study Design

The current study was a prospective cohort study conducted in the Hospital das Clínicas Intensive Care Unit (University of São Paulo, Brazil). Blood samples were collected from six aged septic patients (age range 65–78 years old), six young adult septic patients (age range 22–35 years old), six healthy aged volunteers (age range 60–82 years old), and six healthy young individuals (age range 20–35 years old). All sepsis cases were from patients with clinical illness and did not include patients admitted for trauma or surgical reasons. The majority of patients included in this study were admitted with sepsis, stroke, altered levels of consciousness, pulmonary edema, and asthma and/or chronic obstructive pulmonary disease. Patients who were less than 18 years old, pregnant, HIV-positive, or in end-of-life conditions were excluded. Patients with disseminated malignancies or advanced hepatic disease, those receiving chemotherapy, and those who refused to participate in this study were also excluded. Septic shock was defined according to the criteria of the American College of Chest Physicians/Society of Critical Care Medicine (ACCP/SCCM) Consensus Conference Committee proposed in 1992 [42].

The study protocol was approved by the Hospital das Clínicas Ethics Committee. Patients (or their close relatives) received detailed explanations and provided written consent prior to inclusion in the study (HCFMUSP Protocol # 1207/09).

### 4.2. Oligoarray Reannotation for the lncRNA Analysis

The commercial oligoarray used in the gene expression experiments (Agilent DNA SurePrint G3 Human Gene Expression 8x60k v2 Oligoarray, design ID # 039494; Agilent, Santa Clara, CA, USA) contains 58,717 probes of which 36,075 interrogate mRNAs, 14,450 interrogate known or putative lncRNAs, plus 141 QC control probes. In addition, 5624 probes are poorly annotated (i.e., it is unclear which transcript evidence was used for probe design), and 2568 have no annotation at all. A reannotation of the array was performed as follows. The BLAT tool [43] was used to align all probes to the human genome (version GRCh37). Alignments with up to 2 mismatches and gapped alignments due to RNA splicing were accepted. Probes that aligned with more than 4 genomic coordinates where excluded from further analysis. Genomic coordinates of the remaining probes (“approved probes”) were cross-referenced to different gene annotation databases: GENCODE [44], Broad Institute Human lncRNAs [45], LNCipedia [46], and NONCODE [47]. For probes that matched more than one database, the annotation preference was given according to the following order of priority: GENCODE > Broad Institute > LNCipedia > NONCODE.

As some probes were aligned to regions with more than one annotation type, hierarchical classification criteria were adopted as follows:

1. If a probe aligned to exons of protein-coding genes, it was annotated as “protein-coding”.
2. If a probe aligned to annotated exons of RNAs classified as any pseudogene, and did not overlap protein-coding exons, it was annotated as “pseudogene”.
3. If a probe aligned to annotated exons of lncRNAs and was not previously classified as a protein-coding or pseudogene, it was classified as “lncRNA”.
4. If a probe aligned only to an intron of an annotated gene, to regions in the opposite strand of a known gene, or to regions without any gene annotations, in either strand, it was classified as “poorly annotated RNA”.

A summary of the reannotation results is shown as Supplementary Material (Figure S1). The expression data and probe reannotation information are deposited at the Gene Expression Omnibus (accession number GSE89376 associated to platform GPL22628).

### 4.3. RNA Extraction, Oligoarray Hybridization, and Data Pre-Processing

Sample RNA isolation, target labeling, and hybridization to expression oligoarrays were described in detail in a previous publication reporting an analysis of global expression profiles of protein-coding genes in sepsis [5].

Twenty-four blood samples (six young adults with sepsis, six control young adults, six elderly patients with sepsis, and six control elderly) were processed immediately after collection. The anticoagulant-treated blood was layered on the Ficoll-Paque PLUS solution (GE Healthcare, Chicago, IL, USA) and centrifuged for a short period of time. Differential migration during centrifugation results in the formation of layers containing different cell types. The bottom layer contains erythrocytes that have been aggregated by the Ficoll and, therefore, sediment completely through the Ficoll-Paque PLUS. The layer immediately above the erythrocyte layer contains the granulocytes, which, at the osmotic pressure of the Ficoll-Paque PLUS solution, attain a density great enough to migrate through the Ficoll-Paque PLUS layer. After Ficoll-Paque PLUS density gradient centrifugation, we separated the second layer containing the granulocytes. This layer was transferred to new tubes, diluted in lysis buffer and kept on ice for 10 min. After centrifugation at 290× *g* for 10 min at 4 °C, the pellet was resuspended in lysis buffer and kept on ice for an additional 10 min. A new centrifugation step was performed at 2500× *g* for 2 min at room temperature and the samples were washed with phosphate-buffered saline (PBS). Finally, the samples were centrifuged at 1500× *g* for 2 min at room temperature and the pellet was resuspended in Trizol (Life Technology, Carlsbad, CA, USA) and stored at −80 °C. Total RNA was isolated using Trizol reagent following the manufacturer’s protocol and its integrity and concentration were assessed using the Agilent 2100 Bioanalyzer and the RNA 6000 Nano Kit (Agilent Technologies). Expression levels of both protein-coding and lncRNAs were evaluated using the SurePrint G3 Human Gene Expression 8x60K v2 oligoarray and the Low Input Quick Amp Labeling kit, following a two-color labeling protocol (Agilent Technologies). Cyanine-3 (Cy3)-labeled RNA from each patient sample and cyanine-5 (Cy5)-labeled reference RNA (Universal Human Reference RNA, Agilent, cat. #740000) were combined and hybridized to individual oligoarrays following the manufacturer’s protocol.

Data acquisition and pre-processing of oligoarray expression data is described in detail in [5]. Briefly, oligoarrays were scanned using the SureScan Microarray Scanner (Agilent Technologies) and images were processed using the Feature Extraction Software v12 (Agilent Technologies) for quality control, determination of feature intensities and ratios, and for background correction. We considered for further analysis oligoarray normalized features (Cy3/Cy5 ratios) that were consistently expressed, (i.e., “detected” well-above background (WAB) in at least 5 out 6 subjects in at least one sample group).

### 4.4. Hierarchical Clustering of lncRNAs

Unsupervised hierarchical clustering of expression measurements from lncRNAs detected in septic and control patients (top 5% with higher coefficient of variation) was performed using UPGMA clustering and Pearson correlation as a distance measurement in the Spotfire analysis software (Tibco Inc., Palo Alto, CA, USA).

### 4.5. Detection of Differentially-Expressed Genes

Genes differentially expressed in sepsis (DEGs) were identified as described previously [48]. Briefly, genes were considered as DEGs when detected by two statistical methods, namely Significance Analysis of Microarrays [49] and Rank product [50]. To limit the number of false-positives, we only considered for further analysis DEGs with a *p* value ≤ 0.01 by both methods. Gene measurements are reported as average expression ratios between sepsis and control samples.

### 4.6. Building Co-Expression Networks in Sepsis

We employed a weighted correlation network analysis (WGCNA) implemented as a package in R [22] to construct co-expression networks with protein-coding mRNAs and ncRNAs. In the gene co-expression networks built by WGCNA, each node is linked to all other nodes but with variable strength. The connection strength is the absolute value of the Pearson correlation raised by the power of a β constant that assigns greater weight to values closer to 1 in exchange for a possible loss of information. This adjusted correlation measurement will be referred hereafter as “network similarity”, and those node pair similarity measurements are the basis to calculate the topological overlap matrix (TOM), which measures node interconnectivity, ranging from 1 (nodes that are identically connected to all other nodes) to 0 (nodes that are not mutually connected to any other node). Each gene is assigned a connectivity measurement that describes how central (or hub) the gene is relative to a given network, information from which we can infer that its expression exerts some kind of influence on the connected genes [22]. Here, we used WGCNA to create two networks, one with expression data from the 12 samples from elderly subjects (septic and controls), and the other with data from the 12 samples from young adult (septic and controls). In both cases, the networks were built with gene expression from all genes that passed the pre-processing filtering step (see above). For both networks, the β exponential constant factor to adjust the correlation was set to 13.

For each lncRNA, the average network similarity to all protein-coding genes in a given pathway was compared to the average network similarities of each member within the pathway. If the average network similarity of the lncRNA was greater than the median similarity measured within the pathway, this ncRNA displays more network similarity to that pathway than half of its annotated members. This indicates a strong pathway interaction, and we used this criterion to select the most relevant lncRNA–protein-coding nodes in the sepsis co-expression networks.

### 4.7. Functional Annotation and Pathway Analysis

Genes with higher connectivity in co-expression networks from elderly or young adult subjects were sorted by connectivity and analyzed to search for the enrichment of particular gene categories and molecular pathways using the gProfiler tool [51]. A list with all detected probes was provided as background for the gene enrichment analysis. Only terms with an enrichment *p* value < 0.05 and that contained more than four genes were further considered.

## Figures and Tables

**Table 1:** Classification of array probes after the re-annotation procedure. Criteria for probe approval, detection and selection of differentially expressed genes (*p* value ≤ 0.01) in each experimental group according to disease status or age are detailed in the Materials and Methods section.

Differentially Expressed Genes (DEGs)						
			Elderly vs. Adults		Sepsis vs. Controls	
	Total	Detected	Sepsis	Control	Elderly	Adults
In the array	50,599	15,612	37	69	1677	1862
Approved	47,012	14,264	36	65	1411	1615
Protein-coding	26,542	11,895	27	54	1121	1353
Pseudogenes	2869	834	2	5	151	128
lncRNAs	14,832	1185	5	4	114	99
Poorly annotated	2781	350	2	2	25	36

**Table 2:** Selected long noncoding RNAs (lncRNAs) among the most connected (top 15%) DEGs in co-expression networks from both elderly and adult subjects that show an average local similarity greater than the median similarity of enriched pathways related to protein translation. All these five lncRNAs are transcribed from ribosomal protein pseudogenes. The average fold-changes (FCs) between sepsis and control subjects for each age group are shown. Significant *p* values are in bold.

Sepsis vs. Control							
Gene	Connectivity		Type	Elderly		Adults	
	Elderly	Adults		FC	*p* Value	FC	*p* Value
*RP11-302F12.1*	774	572	pseudogene	0.24	**7.5 × 10^−5^**	0.35	**1.1 × 10^−3^**
*RP3-486D24.1*	746	581	pseudogene	0.36	**6.0 × 10^−4^**	0.43	**3.0 × 10^−3^**
*RPL13AP7*	735	631	pseudogene	0.40	**2.8 × 10^−3^**	0.43	**1.5 × 10^−3^**
*RP11-159C21.4*	676	483	pseudogene	0.39	**2.1 × 10^−3^**	0.42	**8.8 × 10^−3^**
*RP11-179H18.5*	660	566	pseudogene	0.35	**6.0 × 10^−4^**	0.38	**2.1 × 10^−3^**

**Table 3:** Selected lncRNAs among the most differentially connected (top 20%) DEGs between co-expression networks from elderly and adults that show an average local similarity greater than the median similarity of enriched pathways related to cellular respiration in at least one network, and show an inverted expression pattern between networks. The average fold-changes (FCs) between sepsis and control subjects for each age group are shown. Significant *p* values are in bold.

Sepsis vs. Control							
Gene	Connectivity		Type	Elderly		Adults	
	Elderly	Adults		FC	*p* Value	FC	*p* Value
*RP11-383M4.6*	15.0	450	lincRNA	0.97	7.4 × 10^−1^	1.86	**3.2 × 10^−3^**
*CTC-293G12.1*	10.8	414	lincRNA	0.94	5.2 × 10^−1^	1.95	**5.2 × 10^−3^**
*lnc-THUMPD3-1*	3.7	397	ncRNA	0.94	7.2 × 10^−1^	2.25	**9.7 × 10^−4^**
*RP11-121L11.3*	16.9	372	lincRNA	0.98	7.8 × 10^−1^	1.86	**4.0 × 10^−3^**
*MYCNOS*	4.0	298	antisense	0.95	6.7 × 10^−1^	1.76	**9.6 × 10^−3^**
*MALAT1*	6.6	281	lincRNA	1.21	5.0 × 10^−1^	0.37	**2.0 × 10^−4^**
*AC010970.2*	7.3	280	pseudogene	0.92	5.8 × 10^−1^	1.86	**4.1 × 10^−3^**
*RPL10P3*	14.9	274	pseudogene	1.11	8.4 × 10^−1^	0.41	**8.0 × 10^−4^**
*SNORD11*	5.6	237	snoRNA	1.25	4.0 × 10^−1^	0.38	**3.5 × 10^−4^**
*RPL13P5*	1.7	235	pseudogene	0.91	8.6 × 10^−1^	2.37	**6.0 × 10^−4^**
*LINC00355*	291	11.0	lincRNA	1.79	**5.3 × 10^−3^**	0.96	7.3 × 10^−1^

## Supplementary Materials

The following are available online at www.mdpi.com/2311-553X/3/1/5/s1, Figure S1: Summary of the reannotation pipeline, Figure S2: Weighted correlation network analysis (WGCNA) gene co-expression networks from elderly and young adults with data from sepsis patients and control subjects, Figure S3: Elderly and adult co-expression networks of ncRNAs and protein-coding mRNAs in molecular pathways enriched among the top 15% most connected or the top 20% most differentially connected genes, Figure S4: Genomic context of lncRNAs differentially expressed in sepsis and displaying high connectivity in gene co-expression networks, Table S1: List with all detected array probes along with their gene type (protein-coding, lncRNA, pseudogene, poorly annotated), gene ID, chromosomal location, relative expression between septic and control samples, associated *p* values.
