# Supplementary material for: Insights into the Function of Long Noncoding RNAs in Sepsis Revealed by Gene Co-Expression Network Analysis
Source: Noncoding RNA. 2017 Jan 26;3(1):5. doi: 10.3390/ncrna3010005 (PMC5831999; doi:10.3390/ncrna3010005)
Supplement: Supplementary file 1 [file ncrna-03-00005-s001.pdf]

Insights into the Function of Long Noncoding RNAs in Sepsis Revealed by Gene Co-Expression Network Analysis

Diogo Vieira da Silva Pellegrina, Patricia Severino, Hermes Vieira Barbeiro, Heraldo Possolo de Souza, Marcel Cerqueira César Machado, Fabiano Pinheiro da Silva and Eduardo Moraes Reis

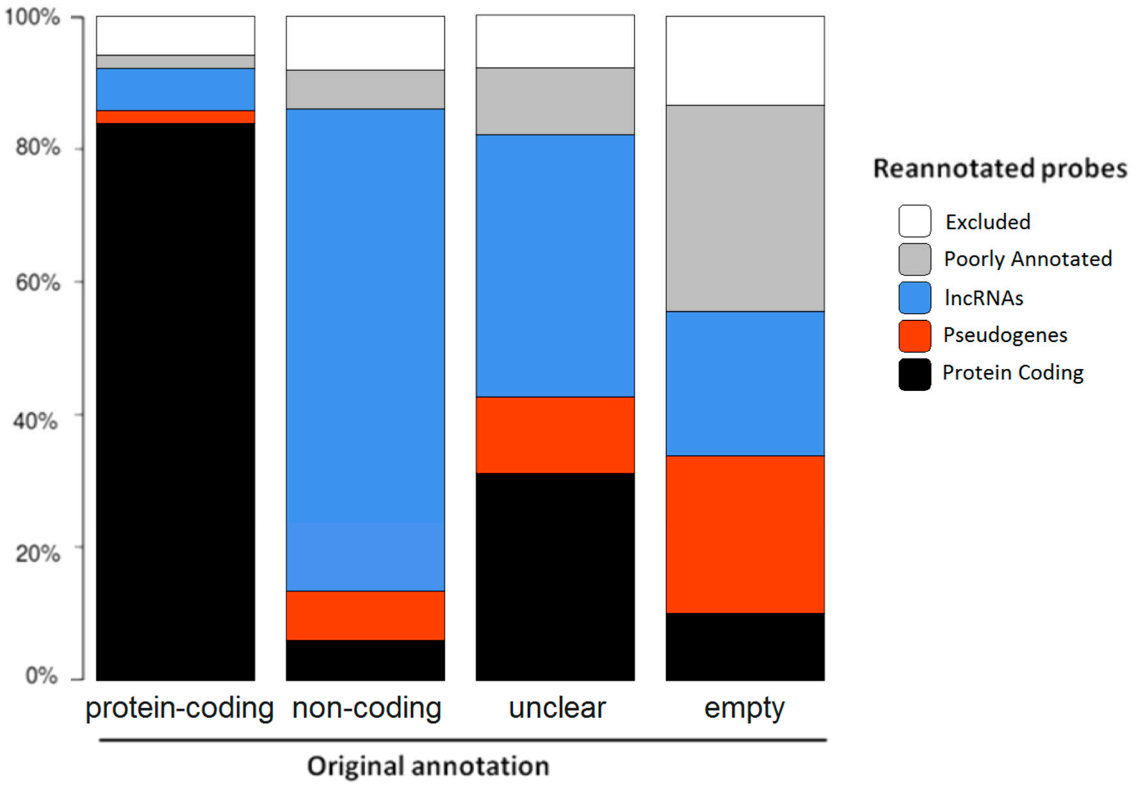

Figure S1. Summary of the reannotation pipeline. The *x* axis summarizes the original probe annotation of the oligoarray used in the experiments (Agilent DNA SurePrint G3 Human Gene Expression 8 × 60 k v2). The "empty" category refers to probes with a blank line in the original Agilent annotation file. The "unclear" category refers to poorly annotated probes, with not enough information to be assigned to a protein-coding or noncoding gene locus. Probes were reannotated following genome mapping and cross-reference with catalogs of protein-coding and noncoding RNAs (GENCODE, Broad Institute Human lincRNA, LNCipedia, NONCODE) as shown in the *y* axis (see Methods for details of the reannotation pipeline). A fraction of the probes aligned to multiple genomic sites (**white** bars) or where poorly annotated (**gray** bars) and were excluded from further analysis. Noncoding transcripts were classified as known lncRNAs or pseudogenes (**blue** and **red** bars, respectively). Protein-coding mRNAs are shown as **black** bars.

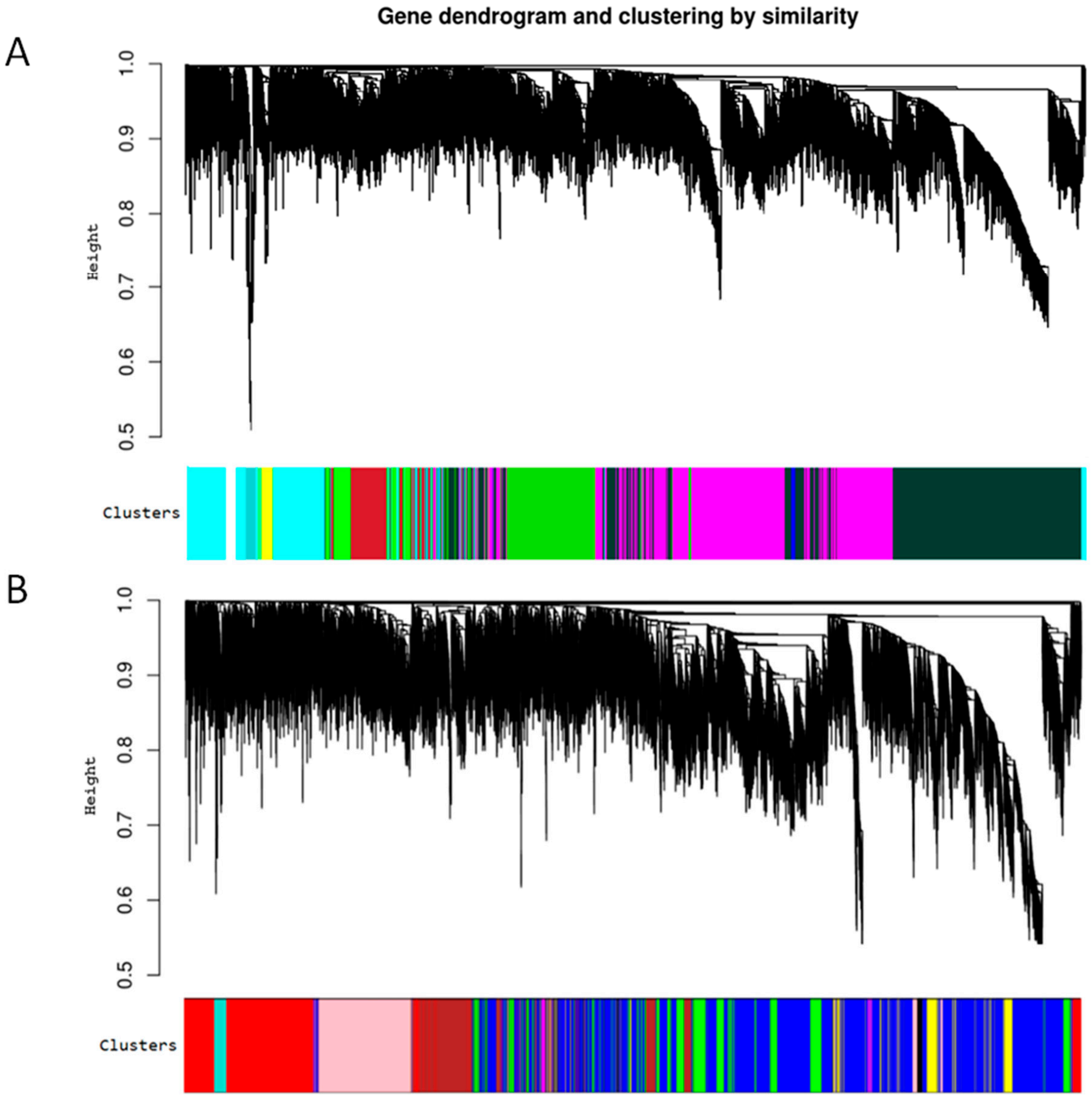

Figure S2. Weighted correlation network analysis (WGCNA) gene co-expression networks from elderly (panel **A**) and young adults (panel **B**) with data from sepsis patients and control subjects. The dendrogram reflects gene clustering based on network similarity. Genes were further clustered by their node interconnectivity as shown in the colored horizontal bars.

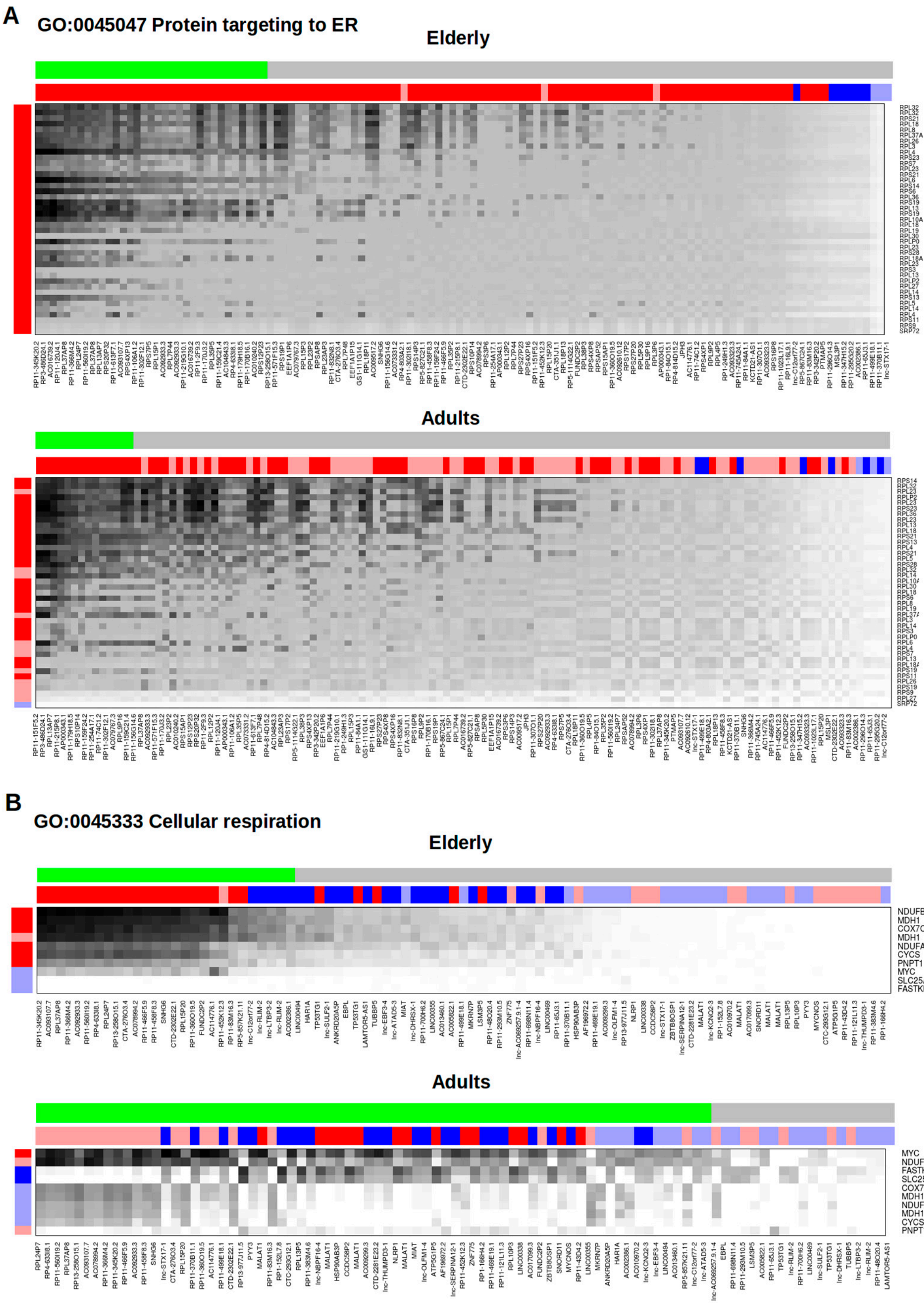

Figure S3. Elderly and adult co-expression networks of ncRNAs (columns) and protein-coding mRNAs (rows) in molecular pathways enriched among the top 15% most connected (panel **A**) or the top 20% most differentially connected (panel **B**) genes. Heatmap colors show the network similarity between each gene pair, with black being the most similar and white the least similar. The gene expression ratios are shown as external bars, where dark blue and red indicate transcripts significantly ($p \leq 0.01$) upregulated or downregulated in sepsis relative to controls, respectively. The lncRNAs with an average local similarity greater than the median similarity of the enriched pathways are indicated in green (see Materials and Methods for details).

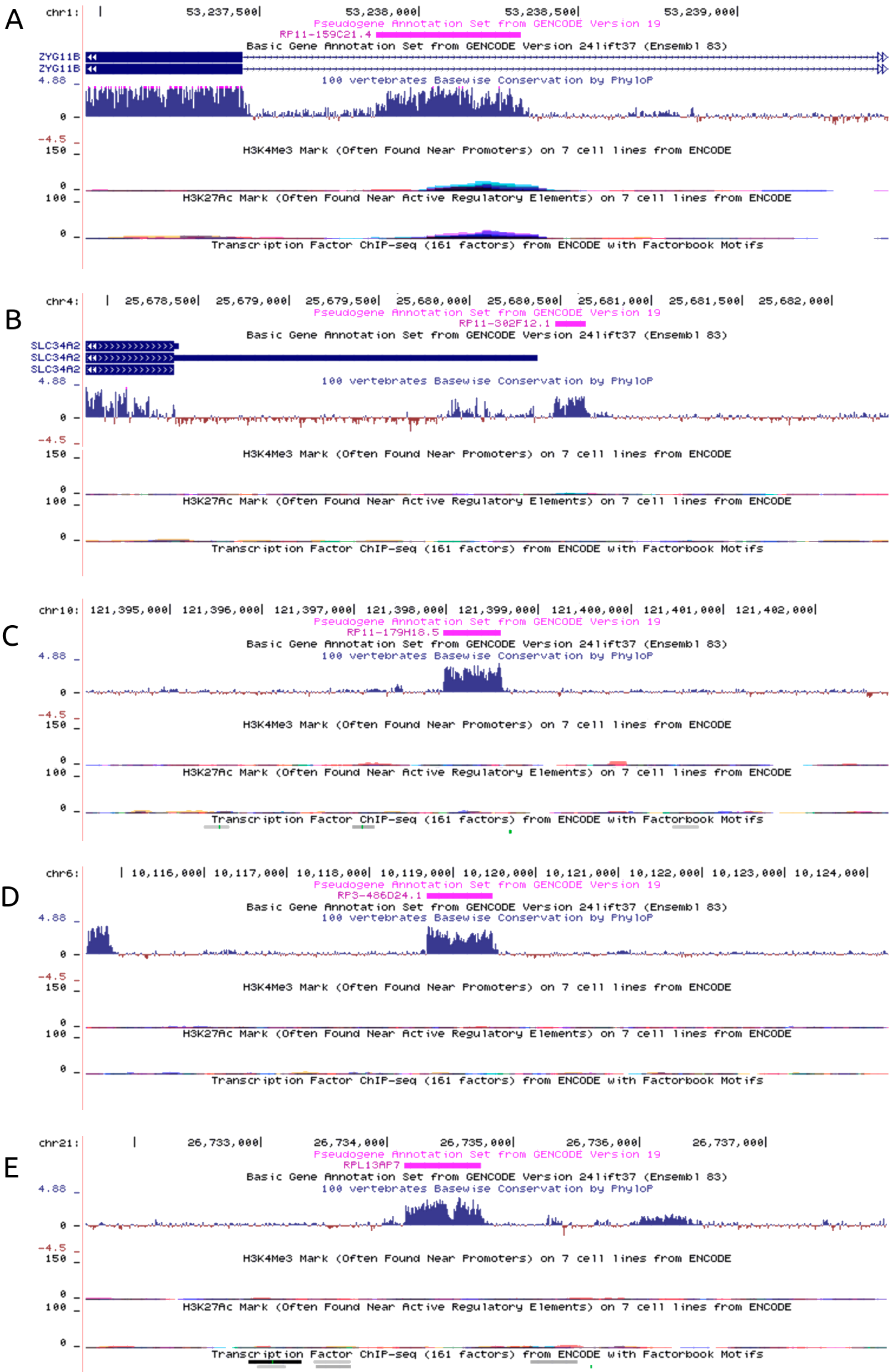
A
chr1:
53,237,500|
53,238,000|
53,238,500|
53,239,000|
Pseudogene Annotation Set from GENCODE Version 19
RP11-159C21.4
Basic Gene Annotation Set from GENCODE Version 24lift37 (Ensembl 83)
ZYG11B
ZYG11B
4.88 _
100 vertebrates Basewise Conservation by PhyloP
0 _
-4.5 _
150 _
H3K4Me3 Mark (Often Found Near Promoters) on 7 cell lines from ENCODE
0 _
100 _
H3K27Ac Mark (Often Found Near Active Regulatory Elements) on 7 cell lines from ENCODE
0 _
Transcription Factor ChIP-seq (161 factors) from ENCODE with Factorbook Motifs
B
chr4:
25,678,500|
25,679,000|
25,679,500|
25,680,000|
25,680,500|
25,681,000|
25,681,500|
25,682,000|
Pseudogene Annotation Set from GENCODE Version 19
RP11-302F12.1
Basic Gene Annotation Set from GENCODE Version 24lift37 (Ensembl 83)
SLC34A2
SLC34A2
SLC34A2
4.88 _
100 vertebrates Basewise Conservation by PhyloP
0 _
-4.5 _
150 _
H3K4Me3 Mark (Often Found Near Promoters) on 7 cell lines from ENCODE
0 _
100 _
H3K27Ac Mark (Often Found Near Active Regulatory Elements) on 7 cell lines from ENCODE
0 _
Transcription Factor ChIP-seq (161 factors) from ENCODE with Factorbook Motifs
C
chr10:
121,395,000|
121,396,000|
121,397,000|
121,398,000|
121,399,000|
121,400,000|
121,401,000|
121,402,000|
Pseudogene Annotation Set from GENCODE Version 19
RP11-179H18.5
Basic Gene Annotation Set from GENCODE Version 24lift37 (Ensembl 83)
4.88 _
100 vertebrates Basewise Conservation by PhyloP
0 _
-4.5 _
150 _
H3K4Me3 Mark (Often Found Near Promoters) on 7 cell lines from ENCODE
0 _
100 _
H3K27Ac Mark (Often Found Near Active Regulatory Elements) on 7 cell lines from ENCODE
0 _
Transcription Factor ChIP-seq (161 factors) from ENCODE with Factorbook Motifs
D
chr6:
10,116,000|
10,117,000|
10,118,000|
10,119,000|
10,120,000|
10,121,000|
10,122,000|
10,123,000|
10,124,000|
Pseudogene Annotation Set from GENCODE Version 19
RP3-486D24.1
Basic Gene Annotation Set from GENCODE Version 24lift37 (Ensembl 83)
4.88 _
100 vertebrates Basewise Conservation by PhyloP
0 _
-4.5 _
150 _
H3K4Me3 Mark (Often Found Near Promoters) on 7 cell lines from ENCODE
0 _
100 _
H3K27Ac Mark (Often Found Near Active Regulatory Elements) on 7 cell lines from ENCODE
0 _
Transcription Factor ChIP-seq (161 factors) from ENCODE with Factorbook Motifs
E
chr21:
26,733,000|
26,734,000|
26,735,000|
26,736,000|
26,737,000|
Pseudogene Annotation Set from GENCODE Version 19
RPL13AP7
Basic Gene Annotation Set from GENCODE Version 24lift37 (Ensembl 83)
4.88 _
100 vertebrates Basewise Conservation by PhyloP
0 _
-4.5 _
150 _
H3K4Me3 Mark (Often Found Near Promoters) on 7 cell lines from ENCODE
0 _
100 _
H3K27Ac Mark (Often Found Near Active Regulatory Elements) on 7 cell lines from ENCODE
0 _
Transcription Factor ChIP-seq (161 factors) from ENCODE with Factorbook Motifs

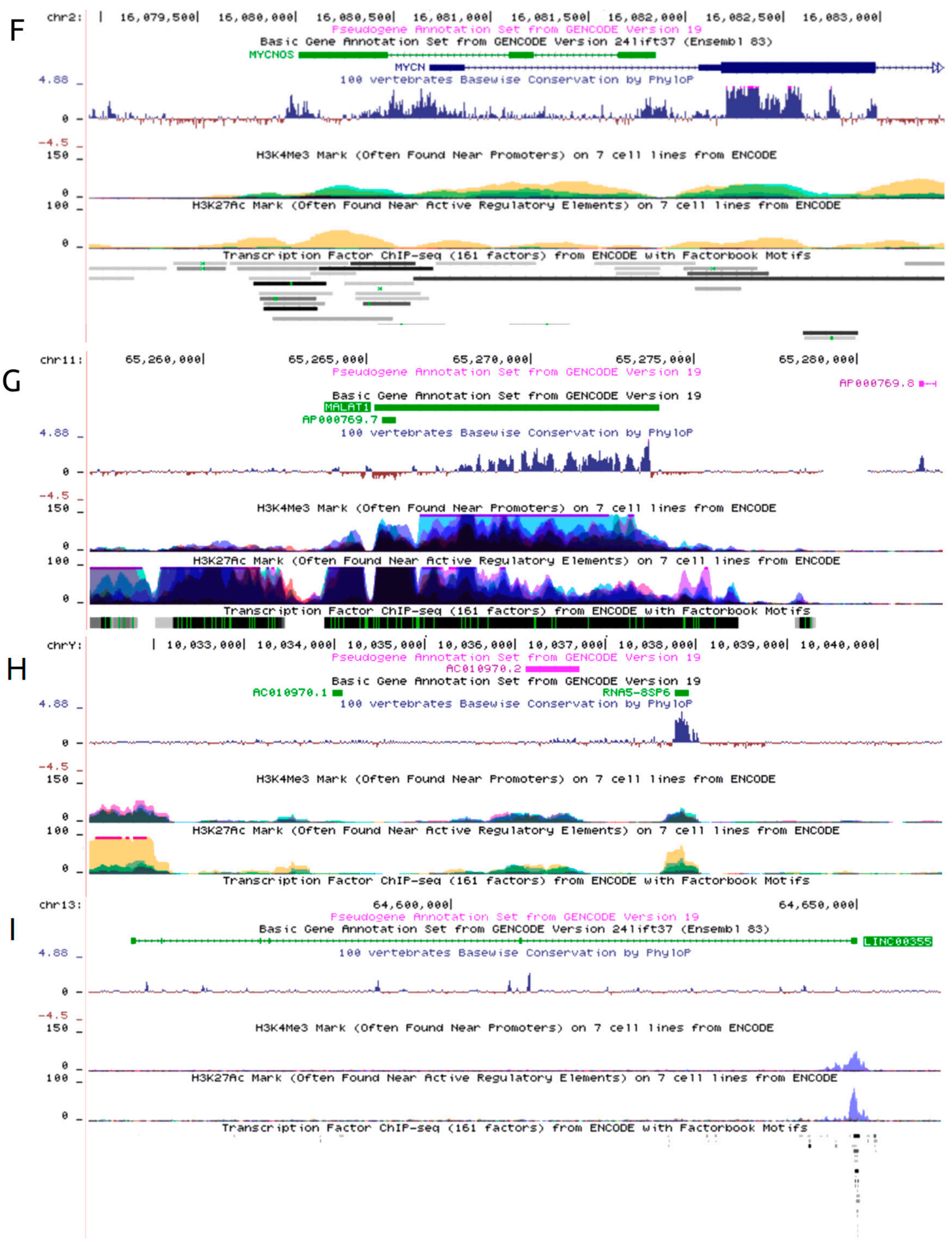

Figure S4. Genomic context of lncRNAs differentially expressed in sepsis and displaying high connectivity in gene co-expression networks. Evolutionary sequence conservation and putative regulatory DNA elements (promoter-associated H3K4me3, enhancer-associated H3k27ac, transcription factor binding sites) are shown. Panels **A**–**E** show the pseudogenes from ribosomal proteins selected among the 15% top connected DEGs. They are all mono-exonic and are all highly conserved; Panels **F**–**H** show lncRNAs selected among the 20% to differentially connected DEGs. (**F**) MYCYNOS; (**G**) AC010970.2; (**H**) MALAT1; (**I**) LINC00355.
